# Local drug delivery systems for the periodontal microenvironment: from barrier penetration to multilevel synergy

**DOI:** 10.3389/fphar.2026.1883391

**Published:** 2026-08-10

**Authors:** Huilin An, Qinxiao Lin, Mengru Zhai, Pengcen Zhou, Zheng Zhang

**Affiliations:** 1 Tianjin Stomatological Hospital and Department of Stomatology, School of Medicine, Nankai University, Tianjin, China; 2 Tianjin Key Laboratory of Oral and Maxillofacial Function Reconstruction and Stomatology, Institute of Nankai University, Tianjin, China

**Keywords:** clinical translation, local drug delivery systems, periodontal microenvironment, periodontitis, programmed release, theranostics

## Abstract

Periodontitis is a chronic inflammatory disease characterized by progressive destruction of periodontal supporting tissues. Although local drug delivery systems (LDDSs) enable site-specific therapy, their clinical efficacy remains limited by the dynamic and heterogeneous periodontal microenvironment. This review systematically delineates multiscale barriers to effective delivery, including biofilm resistance, gingival crevicular fluid (GCF)-mediated clearance, immune-mediated degradation, and impaired tissue regeneration. In response, LDDS design has evolved from passive carriers to multilevel synergistic systems, integrating responsiveness to multiple stimuli and programmed release that aligns with the antibacterial–anti-inflammatory–regenerative sequence. The emergence of theranostic platforms further enables real-time monitoring and feedback-controlled intervention. Despite these advances, a significant translational gap persists. Current models fail to recapitulate the polymicrobial and chronic nature of periodontitis, while overlooked *in vivo* phenomena, including protein corona formation and the reactive oxygen species (ROS) paradox, may compromise therapeutic outcomes. Future strategies should prioritize physiological relevance and precise spatiotemporal regulation to facilitate clinical translation of LDDSs.

## 1 Introduction

Periodontitis is recognized as a chronic, multifactorial inflammatory disease initiated by dental plaque biofilms. It is characterized by the progressive destruction and loss of periodontal supporting tissues, including the gingiva, periodontal ligament, cementum, and alveolar bone. Clinically, the disease presents with gingival bleeding, clinical attachment loss, periodontal pocket formation, and alveolar bone resorption. In advanced stages, tooth mobility and eventual tooth loss may occur, with consequent impairment of masticatory function and esthetics (Papapanou et al., 2018). Accumulating evidence suggests that periodontitis may contribute to low-grade systemic inflammation and disruption of immune homeostasis. It has also been associated with a range of systemic conditions, including cardiovascular disease, type 2 diabetes, Alzheimer’s disease, and adverse pregnancy outcomes (Hajishengallis and Chavakis, 2021; Kamalabadi et al., 2023). Periodontitis is highly prevalent and remains a major challenge to global public health. According to data from the Global Burden of Disease study, the global prevalence of severe periodontitis increased by 91.5% between 1990 and 2021, making it the sixth most common disease worldwide. Both the prevalence and severity of periodontitis increase with age, thereby imposing a substantial burden on healthcare systems (“Trends in the global, regional, and national burden of oral conditions from 1990 to 2021: a systematic analysis for the Global Burden of Disease Study 2021,” GBD 2021 Oral Disorders Collaborators, 2025).

### 1.1 Treatment of periodontitis

The core objectives of periodontitis treatment are to remove dental plaque biofilm and to control local inflammation. Clinical management primarily relies on mechanical debridement. This includes supragingival scaling, subgingival scaling and root planing (SRP), which are used to remove plaque biofilm and calculus. Although SRP remains the gold standard for the nonsurgical management of periodontitis and can significantly improve clinical outcomes (Sanz et al., 2020), accumulating clinical evidence indicates that mechanical debridement alone is often insufficient to achieve complete eradication of infection within the complex periodontal microenvironment. A systematic review and meta-analysis encompassing 27 prospective studies demonstrated that approximately 11.7% of tooth sites retained residual deep periodontal pockets (probing pocket depth [PPD] ≥ 5 mm) following nonsurgical periodontal therapy, with patients presenting an average of 14 residual deep periodontal pockets (Citterio et al., 2022).

These residual periodontal pockets not only serve as critical niches for the recolonization of periodontal pathogens but also represent significant predictors of continued disease progression and ongoing clinical attachment loss. Collectively, these findings suggest that although conventional mechanical debridement effectively reduces the microbial burden, it is insufficient to completely eliminate persistent infectious foci, thereby compromising long-term therapeutic outcomes. In moderate-to-severe or aggressive periodontitis, adjunctive systemic antibiotics have been shown to improve the short-term outcomes of SRP (Zhang et al., 2016). This effect appears to be more pronounced in extensive disease, younger patients, and individuals with systemic predisposing factors (Nakajima et al., 2025; Sulugodu Ramachandra et al., 2025). However, a systematic review by Garcia Canas et al. reported that, although adjunctive systemic antibiotics generally improved key clinical parameters, including probing pocket depth (PPD), clinical attachment level (CAL), and bleeding on probing (BOP), the substantial heterogeneity among the available studies precluded definitive clinical recommendations. Moreover, the therapeutic benefits appeared to be largely confined to selected patient populations. Consequently, systemic antibiotic therapy cannot yet be considered a routine long-term treatment strategy for all patients with periodontitis (Garcia Canas et al., 2015). In addition, systemic administration is inherently associated with several limitations. Drug concentrations within periodontal pockets are often insufficient to achieve and maintain bactericidal levels. Furthermore, prolonged or high-dose systemic antibiotic therapy may increase the risk of systemic adverse effects, gut microbiota dysbiosis, and the emergence of antimicrobial resistance (Lima de Sousa et al., 2024; Viglianisi et al., 2023).

### 1.2 Local drug delivery systems: overview and limitations

LDDSs have emerged as an adjunct to periodontal nonsurgical treatment, allowing drugs to be delivered directly into periodontal pockets. This approach increases local drug exposure while reducing systemic adverse effects (Sha et al., 2025). Depending on the therapeutic agents used, LDDSs can provide antibacterial, anti-inflammatory, and tissue-reparative effects. However, their efficacy within the complex periodontal microenvironment remains constrained by multiple factors.

An ideal delivery system is expected to maintain sustained drug release over several days to weeks (Mirzaeei et al., 2021). Nevertheless, conventional LDDSs generally exhibit limited control over drug release kinetics and are particularly prone to an undesirable initial burst release. Extensive evidence has demonstrated that this rapid release results in the premature depletion of a substantial proportion of the loaded drug, thereby hindering the maintenance of therapeutic drug concentrations required for sustained treatment. For instance, in an *in vitro* release study, the chlorhexidine-loaded gelatin hydrogel developed by Zussman et al. released approximately 39% of its payload within the first 2 h, followed by a marked decline in the release rate. Although drug release was sustained for approximately 6 days, the amount released during the later stage was substantially reduced (Zussman et al., 2022). Therefore, local drug concentrations typically reach a peak shortly after administration but decline rapidly thereafter, making it difficult to maintain therapeutic levels throughout the entire treatment course. This inadequate temporal control over drug availability is considered one of the major factors limiting the long-term therapeutic efficacy of conventional LDDSs (Amato et al., 2023; Marzaman et al., 2025; Nakajima et al., 2025). In addition, structural instability may limit drug-loading capacity, thereby reducing the effective dose delivered (Lei et al., 2010).

Continuous gingival crevicular fluid flow, saliva washout, and mechanical forces from mastication further promote dilution and clearance of the delivery system, thereby shortening local retention time (Lima de Sousa et al., 2024; Ramburrun et al., 2025). Recent studies indicate that the continuous fluid shear stress within periodontal pockets, together with the dynamic mechanical environment of the oral cavity, can markedly reduce the retention time of conventional LDDSs, resulting in their rapid clearance before adequate drug diffusion is achieved and collectively limiting local drug exposure. Early investigations into bioadhesive delivery systems for periodontal applications demonstrated that chitosan-based gels with superior bioadhesive properties exhibited significantly prolonged retention within periodontal pockets compared with conventional gels, whereas non-bioadhesive hydrogels were more readily displaced by the continuous flushing action of gingival crevicular fluid (GCF). These findings highlight bioadhesion as a critical determinant of the therapeutic performance of local drug delivery systems (Needleman et al., 1997). These effects are more pronounced when materials lack sufficient adhesion or cannot be effectively retained at the site of application (Oschatz et al., 2025). Inspired by the remarkable wet adhesion mechanism of mussels, increasing efforts have been devoted to incorporating catechol and other wet-adhesive moieties into LDDSs to enhance their tissue adhesion under the moist conditions of the oral cavity. For example, Xu et al. developed a catechol-functionalized chitosan hydrogel that prolonged the adhesion time on moist oral mucosa from approximately 1.5 h for unmodified chitosan to more than 6 h. These findings further demonstrate that enhancing tissue adhesion represents an effective strategy for prolonging local drug retention and improving therapeutic efficacy (Xu et al., 2015).

Even after achieving prolonged local retention, therapeutic agents must still penetrate the dental biofilm, whose dense extracellular polymeric substances (EPS) matrix constitutes a formidable diffusion barrier. A substantial body of evidence has demonstrated that the EPS matrix not only acts as a physical barrier that impedes the penetration of antimicrobial agents and nanocarriers into the deeper layers of the biofilm, but also reduces their effective intra-biofilm concentrations. As a consequence, bacteria residing in the deeper biofilm layers remain exposed to subinhibitory drug concentrations, thereby promoting persistent infection and increasing the risk of disease recurrence (Koo et al., 2017; Stewart, 2015). A seminal study by Stewart et al. on biofilm diffusion further demonstrated that mature biofilms substantially impede the diffusion of antimicrobial agents, thereby limiting their access to the deep anaerobic bacterial communities embedded within the biofilm (Stewart, 1996). For example, Kang et al. developed a magnetically responsive nanodelivery system and demonstrated, using a multispecies periodontal biofilm model, that conventional antimicrobial formulations exhibited limited bactericidal activity against bacteria residing in the basal regions of the biofilm owing to their poor penetration capability. In contrast, magnetic field-assisted penetration enhanced nanoparticle transport into the deeper biofilm layers, resulting in improved eradication of deeply embedded bacteria. These findings further underscore the biofilm barrier as a major obstacle limiting the therapeutic efficacy of LDDSs (Kang et al., 2024). As drug concentrations decline, residual bacteria may survive and repopulate, increasing the risk of recurrence (Karmakar et al., 2023; Sha et al., 2025; Viglianisi et al., 2023).

Beyond microbial penetration and retention, dysregulation of the host immune response also determines whether local delivery can produce durable therapeutic benefits. Increasing evidence suggests that, even when the bacterial burden is effectively controlled, persistent host immune dysregulation continues to drive chronic inflammation and alveolar bone resorption. Hajishengallis et al. proposed that periodontitis is fundamentally a chronic inflammatory disease driven by the interplay between microbial dysbiosis and host immune dysfunction (Hajishengallis, 2015). Therefore, therapeutic strategies that rely solely on broad-spectrum antimicrobial activity are often insufficient to restore local immune homeostasis. Conventional LDDSs largely rely on broad-spectrum antimicrobial strategies and provide limited modulation of key pathogenic pathways. Such approaches may disrupt the local microbiome and are often insufficient to restore the inflammatory microenvironment, thereby limiting long-term efficacy (Nakajima et al., 2025; Ramburrun et al., 2025).

## 2 Targets of LDDSs in the periodontal microenvironment

**FIGURE 1:**
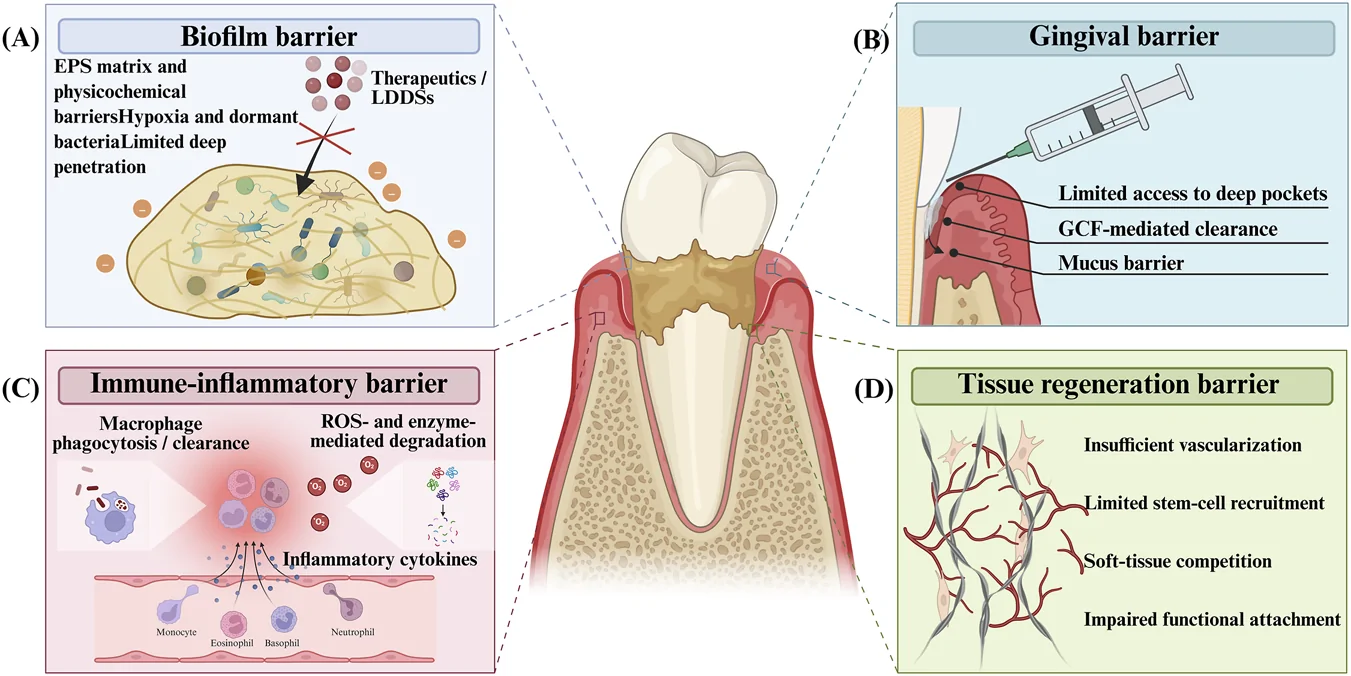
Multiscale barriers limiting periodontal local drug delivery. **(A)** The biofilm barrier restricts the penetration and distribution of therapeutics because of the extracellular polymeric substance (EPS) matrix, physicochemical sequestration, and metabolic heterogeneity within deep biofilm layers. **(B)** The gingival barrier limits local delivery through restricted access to deep periodontal pockets, gingival crevicular fluid (GCF)-mediated clearance, and the mucus/epithelial barrier. **(C)** The immune-inflammatory barrier compromises delivery performance through macrophage phagocytosis, inflammatory cytokines, and ROS-/enzyme-mediated material degradation. **(D)** The tissue regeneration barrier is characterized by insufficient vascularization, limited stem-cell recruitment, soft-tissue competition, and impaired bone–PDL–cementum regeneration. Collectively, these barriers constrain the therapeutic efficacy of periodontal local drug delivery systems (LDDSs).

**FIGURE 2:**
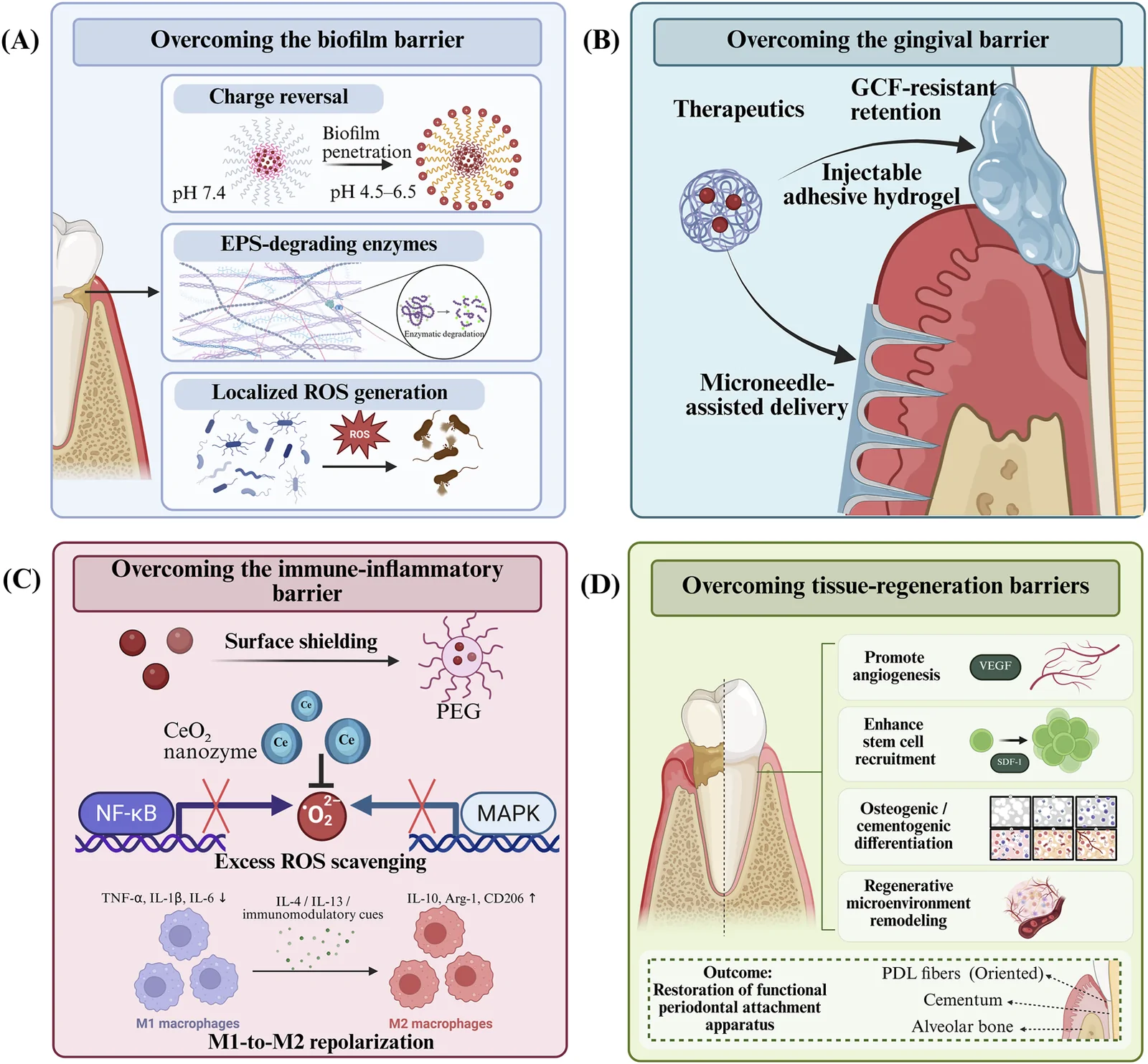
Barrier-overcoming strategies for periodontal local drug delivery systems. **(A)** Representative strategies for overcoming the biofilm barrier include pH-responsive charge reversal, EPS-degrading enzymes, and localized ROS-mediated antibacterial therapy to enhance biofilm penetration and disruption. **(B)** Strategies for overcoming the gingival barrier include adhesive hydrogels and microneedle-assisted delivery to improve tissue access, wet retention, and resistance to GCF-mediated clearance. **(C)** Strategies for overcoming the immune-inflammatory barrier include surface shielding, excess ROS scavenging, inhibition of inflammatory signaling pathways, and macrophage reprogramming to reduce immune clearance and inflammatory damage. **(D)** Strategies for overcoming tissue-regeneration barriers include promoting angiogenesis, enhancing stem-cell recruitment, inducing osteogenic/cementogenic differentiation, and remodeling the regenerative microenvironment to facilitate functional periodontal repair.

The therapeutic performance of LDDSs in periodontitis is constrained by the complex and dynamic microenvironment within periodontal pockets. Rather than a single limiting factor, multiple barriers coexist and interact across different biological scales, including microbial biofilms, gingival tissues, host immune responses, and regenerative processes. These barriers not only restrict drug penetration and retention but also interfere with therapeutic efficacy and tissue repair (Figure 1). The corresponding strategies for overcoming these barriers are summarized in Figure 2.

### 2.1 Biofilm barrier

#### 2.1.1 Dental plaque biofilm architecture: a structural basis for treatment resistance in periodontitis

Dental plaque biofilms are recognized as the primary initiating factor in periodontitis. Compared with planktonic bacteria, biofilm-associated bacteria exhibit markedly increased resistance to antimicrobial agents, with minimum inhibitory concentrations (MICs) reported to be 100–1,000 times higher (Abdulkareem et al., 2023). In their pioneering studies on chronic infections, Costerton et al. demonstrated that a large proportion of bacteria within mature biofilms remained viable even when exposed to antimicrobial concentrations far exceeding the MICs. This observation indicates that biofilm-associated tolerance arises primarily from the complex three-dimensional architecture and unique microenvironment of the biofilm, rather than from genetically acquired antimicrobial resistance (Costerton et al., 1999). Therefore, effective antibacterial therapy with LDDSs requires not only successful drug deposition within the periodontal pocket but also the ability to overcome the hierarchical barriers imposed by the biofilm, thereby ensuring sufficient drug penetration into the deeper biofilm layers.

The structural framework of the biofilm is formed by extracellular polymeric substances (EPS) secreted by bacteria, primarily composed of polysaccharides, proteins, lipids, and extracellular DNA (eDNA) (Bowen et al., 2018). EPS forms a locally organized, viscous three-dimensional polymeric network. This network not only restricts the penetration of macromolecular therapeutics and nanocarriers into the biofilm interior but also limits the local accumulation of therapeutic agents within its deeper regions. In a recent comprehensive review of nanodelivery systems, Lv et al. highlighted that the high viscosity, dense architecture, and negative surface charge of the EPS matrix collectively constitute the principal physical barrier to biofilm penetration. These characteristics represent the fundamental cause of the limited penetration efficiency of most LDDSs and have consequently become a primary target for the development of next-generation nanodelivery strategies (Lv et al., 2023).

This concept has been further substantiated by experimental studies in periodontology. Kang et al. established a multispecies periodontal biofilm model comprising five major periodontal pathogens, including *Porphyromonas gingivalis* and *Fusobacterium nucleatum,* and employed scanning electron microscopy (SEM) and confocal laser scanning microscopy (CLSM) to evaluate nanoparticle distribution within the biofilm. The results showed that conventional antimicrobial formulations, owing to their limited ability to penetrate the EPS matrix, primarily eliminated bacteria located in the superficial biofilm layers. In contrast, magnetically responsive nanoparticles, under the guidance of an external magnetic field, successfully penetrated into the basal biofilm layers, significantly enhancing the eradication of deeply embedded bacteria while reducing the thickness of 7-day mature biofilms from 22 μm to 13 μm. Collectively, these findings provide direct evidence that insufficient penetration into the deeper biofilm layers represents a critical factor limiting the therapeutic efficacy of LDDSs (Kang et al., 2024).

Owing to the abundance of carboxyl and phosphate groups within EPS polysaccharides and extracellular DNA (eDNA), the biofilm matrix typically carries a net negative charge. This negatively charged matrix can electrostatically sequester cationic antimicrobial agents, thereby further restricting their diffusion into the deeper biofilm layers. Using fluorescence tracing in combination with confocal laser scanning microscopy (CLSM), Deiss-Yehiely et al. demonstrated that conventionally cationic nanoparticles were readily trapped by the EPS matrix in the superficial biofilm layers. In contrast, nanoparticles engineered with a pH-responsive charge-reversal strategy exhibited enhanced intra-biofilm diffusion and superior overall antibacterial efficacy. These findings further highlight the electrostatic barrier imposed by the EPS matrix as a critical determinant of drug penetration within biofilms (Deiss-Yehiely et al., 2023).

As biofilms progressively mature and thicken, the continuous consumption of oxygen and nutrients by bacteria in the superficial layers gradually establishes pronounced oxygen and nutrient gradients within the biofilm. As a result, the deeper biofilm regions are maintained in a persistently hypoxic, nutrient-deprived, and mildly acidic microenvironment. This hostile microenvironment not only compromises the bactericidal activity of local immune cells but also diminishes the efficacy of certain antimicrobial agents (Stewart and Franklin, 2008). Under persistent hypoxic and nutrient-limited conditions, bacteria residing in the deeper biofilm layers gradually transition into a metabolically inactive or dormant state, giving rise to a subpopulation of persister cells. Lewis et al. demonstrated that, although persister cells constitute only a small fraction of the bacterial population, their extremely low metabolic activity renders them intrinsically tolerant to the vast majority of antibiotics whose bactericidal effects depend on active bacterial growth and cell division. Following the cessation of antimicrobial treatment, these cells can readily resume proliferation, thereby contributing to the persistence of chronic infections and the recurrence of disease (Lewis, 2010).

Therefore, even when LDDSs are capable of partially penetrating the biofilm, their ability to eradicate metabolically inactive persister cells residing in the deeper biofilm layers remains markedly limited. This persistent bacterial reservoir is widely recognized as one of the key mechanisms underlying the chronic recurrence of periodontitis.

#### 2.1.2 Precision strategies to overcome biofilm barriers

To overcome the complex physical and biochemical defense mechanisms of biofilms, the design paradigm of LDDSs has gradually evolved from conventional passive diffusion toward active biofilm penetration. In recent years, substantial efforts have focused on enhancing carrier penetration and local retention, facilitating extracellular polymeric substances (EPS) degradation, and developing metabolism-independent antibacterial strategies. Collectively, these approaches aim to improve drug delivery efficiency while enabling the precise eradication of mature biofilms.

To overcome the electrostatic sequestration of cationic therapeutics by extracellular DNA (eDNA) and acidic polysaccharides within the EPS matrix, charge-switching nanodelivery systems have emerged as a promising strategy. These nanocarriers typically maintain a neutral or slightly negative surface charge under physiological conditions, thereby minimizing nonspecific interactions during systemic circulation and with healthy tissues. Upon exposure to the mildly acidic microenvironment of the periodontal pocket (pH 4.5–6.5), protonation of their surface functional groups induces a charge reversal to a positive state, thereby enhancing electrostatic interactions with the negatively charged EPS matrix (Guo et al., 2020). Deiss-Yehiely et al. developed layer-by-layer assembled nanoparticles with pH-responsive charge-switching properties and evaluated their transport behavior using fluorescence tracing and biofilm penetration assays. The results demonstrated that this platform effectively reduced the electrostatic sequestration of nanoparticles by the EPS matrix in the superficial biofilm layers, thereby facilitating their diffusion into the deeper biofilm regions. Therefore, the delivery efficiency of antimicrobial agents within mature biofilms was significantly enhanced, highlighting the potential of charge-switching nanocarriers to overcome the electrostatic barrier associated with the EPS matrix (Deiss-Yehiely et al., 2023). Nevertheless, the effectiveness of this strategy relies on the establishment of a sufficiently acidic microenvironment within the periodontal pocket. As a result, its responsiveness may vary with local pH fluctuations across different stages of disease progression.

Enhancing carrier diffusion alone is insufficient to fully overcome the biofilm barrier. Accordingly, increasing attention has been directed toward matrix-disrupting strategies that actively dismantle the extracellular polymeric substances (EPS) matrix. Unlike conventional antibacterial approaches that primarily rely on direct bacterial killing, these strategies first compromise the structural integrity of the EPS matrix, thereby increasing biofilm porosity and facilitating the diffusion of antimicrobial agents into the deeper biofilm layers, ultimately improving overall therapeutic efficacy. Representative approaches include biofilm-dispersing enzymes, such as DNase I and Dispersin B, as well as stimulus-responsive nanozymes. In a comprehensive review, Wang et al. highlighted that these strategies effectively alleviate the diffusional barrier imposed by the EPS matrix, enhance nanocarrier penetration within mature biofilms, and represent a major direction in the development of next-generation antibiofilm drug delivery systems (Wang et al., 2023). Despite these promising advances, most enzyme-mediated matrix-disrupting strategies remain confined to *in vitro* investigations, with relatively few original *in vivo* studies conducted in periodontitis models. Furthermore, the inherent susceptibility of enzymes to deactivation, coupled with their short local residence time and limited capacity for sustained therapeutic activity, continues to represent a major barrier to clinical translation.

Beyond enhancing drug penetration, increasing attention has recently been directed toward metabolism-independent antibacterial strategies to overcome therapeutic failure associated with persister bacteria. Conventional antibiotics largely depend on metabolically active bacteria to exert their bactericidal effects, whereas photodynamic therapy (PDT), chemodynamic therapy (CDT), and nanozyme-based platforms eliminate bacteria through the *in situ* generation of reactive oxygen species (ROS), which directly oxidize cellular membranes, proteins, and nucleic acids. By circumventing the requirement for active bacterial metabolism, these strategies enable the effective eradication of mature biofilms and persister cells. As a representative example, Xu et al. developed a periodontal microenvironment-responsive metal–polyphenol nanozyme platform capable of releasing bioactive components in response to inflammatory cues. In addition to its potent antibacterial activity, the platform modulated local oxidative stress and promoted periodontal tissue repair. Both *in vitro* and *in vivo* studies demonstrated that this intelligent delivery system significantly suppressed periodontal pathogens, attenuated inflammatory responses, and enhanced alveolar bone regeneration, highlighting the considerable potential of stimuli-responsive LDDSs for comprehensive periodontal therapy (Xu et al., 2023). It is noteworthy that ROS not only contribute to bacterial eradication but also serve as indispensable signaling molecules involved in host cell proliferation, differentiation, and tissue repair. Increasing evidence suggests that the future development of LDDSs should not focus on the continuous generation or complete elimination of ROS. Instead, greater emphasis should be placed on stimuli-responsive strategies capable of achieving spatiotemporally precise regulation of ROS levels, thereby simultaneously controlling infection while preserving the physiological signaling functions of ROS that are essential for periodontal tissue regeneration. This emerging concept, referred to as the ROS paradox, will be discussed in detail in a subsequent section.

### 2.2 The gingival barrier

#### 2.2.1 Gingival tissue architecture and GCF clearance

As LDDSs enter periodontal lesions, they must first overcome barriers imposed by gingival tissue architecture and pathological changes (Joshi et al., 2016). Following the onset of periodontitis, a pathologically deepened periodontal pocket develops between the tooth and the gingival tissue, forming a narrow, irregular, blind-ended cavity with a single external opening. This complex three-dimensional anatomical architecture not only hinders the homogeneous distribution of therapeutic agents within the lesion but also prevents conventional liquid formulations and rigid particulate carriers from efficiently accessing the deep regions of the periodontal pocket. Consequently, local drug exposure is compromised, ultimately limiting therapeutic efficacy.

This anatomical barrier has been further substantiated by experimental studies. Hu et al. established an experimental model that simulated the local drug delivery environment of the periodontal pocket and evaluated an injectable hydrogel as a model delivery system. Their results showed that, compared with injectable and conformable materials capable of *in situ* filling the periodontal pocket, conventional liquid formulations exhibited limited access to the deeper regions of the pocket, with most of the delivered agents remaining near the pocket entrance. These findings indicate that the complex anatomical architecture of the periodontal pocket alone is sufficient to restrict drug diffusion into the deeper lesion, thereby limiting local drug delivery efficiency (Hu et al., 2023).

In addition to the anatomical architecture of the periodontal pocket, the continuous flow of GCF represents another major physiological barrier that compromises the therapeutic performance of LDDSs. Under healthy conditions, the GCF flow rate is approximately 0.05–0.2 μL per site, whereas it can increase by more than an order of magnitude in periodontitis (Goodson, 2003). Under inflammatory conditions, GCF continuously flows from the base of the periodontal pocket toward the oral cavity, generating persistent fluid shear stress. This continuous fluid flow progressively dilutes and washes away locally delivered therapeutics, thereby shortening their residence time within the periodontal pocket and compromising sustained therapeutic exposure (Amato et al., 2023). Makkar et al. developed a gingival crevice-on-chip microfluidic model that recapitulates the periodontal pocket microenvironment, enabling the first simulation of host–microbiome interactions under continuous GCF flow. Their findings demonstrated that dynamic fluid flow not only influenced biofilm development but also accelerated the clearance of locally delivered agents. These results suggest that conventional static *in vitro* models may overestimate the local retention performance of drug delivery systems and further highlight persistent GCF-mediated clearance as a critical physiological barrier limiting the efficacy of local periodontal drug delivery (Makkar et al., 2023).

In addition, the mucus layer covering the gingival epithelium constitutes another natural biological barrier to local drug delivery. Composed primarily of highly glycosylated mucins, this viscoelastic layer restricts the diffusion of macromolecular therapeutics and nanocarriers into deeper tissues through steric hindrance, electrostatic interactions, and adhesive interactions. Meanwhile, the continuous turnover of the mucus layer, together with local fluid flow, further accelerates the clearance of exogenous particles, making it difficult for locally delivered therapeutics to maintain sufficient tissue exposure. These results indicate that particle size, surface charge, and hydrophilic–hydrophobic properties all profoundly influence nanoparticle transport within mucus. Therefore, the mucus barrier is now recognized as one of the key biological barriers limiting the efficiency of local drug delivery (McCright and Maisel, 2020).

Collectively, the complex anatomical architecture of the periodontal pocket, persistent GCF-mediated clearance, and the protective mucus barrier synergistically restrict the intralesional distribution, local retention, and tissue penetration of LDDSs. These interconnected barriers not only reduce local drug exposure but also impede the maintenance of therapeutically effective drug concentrations, thereby limiting the efficacy of local periodontal drug delivery. This evidence highlights the importance of overcoming these anatomical and physiological barriers in the rational design of next-generation LDDSs.

#### 2.2.2 Strategies for drug retention in gingival tissue

To address the rapid loss of locally delivered therapeutics caused by the complex anatomy of the periodontal pocket and the persistent flushing effect of GCF, the design of LDDSs has progressively shifted from simply increasing drug loading toward enhancing local retention. Current strategies primarily focus on strengthening the interfacial adhesion between biomaterials and periodontal tissues, optimizing the injectability and rheological properties of delivery systems, and incorporating mechanically responsive or mechanically anchored designs. Collectively, these approaches prolong drug residence within the periodontal pocket, thereby maintaining therapeutically effective local drug concentrations and ultimately improving treatment outcomes.

Enhancing the mucoadhesive properties of biomaterials has emerged as one of the most widely adopted strategies for improving local retention in periodontal drug delivery. Natural and biomimetic materials, including chitosan, alginate, and dopamine-functionalized polymers, have been extensively explored for periodontal local drug delivery owing to their excellent biocompatibility and wet-tissue adhesive properties. In particular, the catechol moieties of dopamine mimic mussel adhesive proteins, enabling robust adhesion to gingival tissues under wet conditions through multiple interactions, including hydrogen bonding, metal coordination, and, in some cases, covalent bonding. These interactions enhance the resistance of biomaterials to persistent GCF-mediated clearance, thereby prolonging their retention within the periodontal pocket. As a representative example, Lin et al. developed a self-healing adhesive hydrogel composed of carboxymethyl chitosan and polyaldehyde dextran (CMCS/PDA) and systematically evaluated its tissue adhesive properties, rheological behavior, and drug release profile. The hydrogel exhibited stable adhesion under wet conditions and enabled sustained melatonin release, which significantly promoted cell migration while demonstrating potent antibacterial activity, thereby providing a favorable local microenvironment for periodontal tissue regeneration. Nevertheless, the authors also noted that under severe inflammatory conditions accompanied by markedly increased GCF secretion, interfacial adhesion alone may be insufficient to prevent gradual material detachment. Therefore, the long-term stability of single mucoadhesion-based strategies remains to be further improved in the complex periodontal microenvironment (Lin et al., 2023).

Beyond enhancing tissue adhesion, injectable hydrogels have increasingly emerged as an effective strategy for improving local retention owing to their ability to conform to the complex and irregular anatomy of periodontal pockets. As a representative example, Li et al. developed an injectable self-healing hydrogel based on carboxymethyl chitosan (CMCS) and oxidized carboxymethyl cellulose (OCMC), incorporating magnolol nanoparticles (MAG NPs) and nano-hydroxyapatite (nHA) for the treatment of diabetic periodontitis. The hydrogel exhibited excellent injectability and self-healing capability, enabling complete filling of deep periodontal pockets and irregular alveolar bone defects while achieving sustained MAG release of approximately 95.5% over 72 h. Both *in vitro* and *in vivo* studies demonstrated that the system effectively inhibited the growth of *P. gingivalis*, scavenged excessive reactive oxygen species (ROS), promoted macrophage polarization from the pro-inflammatory M1 phenotype toward the pro-regenerative M2 phenotype, and induced the osteogenic differentiation of periodontal ligament stem cells. These combined effects ultimately resulted in significantly enhanced periodontal tissue repair and alveolar bone regeneration in a diabetic periodontitis model. Nevertheless, the performance of this hydrogel was primarily evaluated under static experimental conditions, and its long-term retention under continuous GCF-mediated clearance and sustained mechanical stimulation remains to be further validated (Li et al., 2026).

To further enhance local retention while enabling stimuli-responsive drug release, thermosensitive *in situ* hydrogels have increasingly attracted attention as promising carriers for periodontal drug delivery. These materials remain as low-viscosity liquids at room temperature, facilitating injection into the narrow and anatomically irregular periodontal pocket. Upon administration, they undergo rapid sol–gel transition in response to physiological temperature, forming a three-dimensional hydrogel network *in situ*, thereby prolonging drug retention and enabling sustained drug release. As a representative example, Gu et al. developed a chitosan/β-glycerophosphate thermosensitive hydrogel co-loaded with erythropoietin (EPO) and FK506 and systematically evaluated its therapeutic performance in an experimental periodontitis model. The hydrogel rapidly underwent *in situ* gelation following administration and achieved sustained drug release. Therefore, it significantly reduced the expression of pro-inflammatory cytokines, including TNF-α, IL-1β, and IL-6, while upregulating osteogenesis-related markers, such as RUNX2, OCN, and COL-I. Furthermore, micro-computed tomography (micro-CT) and histological analyses confirmed enhanced alveolar bone regeneration. Nevertheless, the system still exhibited a certain degree of initial burst release, indicating that further optimization of release kinetics is required to achieve both rapid anti-inflammatory activity and prolonged maintenance of therapeutically effective drug concentrations (Gu et al., 2023).

In recent years, strategies for enhancing local drug retention have evolved beyond simply improving material adhesion toward the development of intelligent delivery platforms capable of integrating multiple physical stimuli. Along this line, Roldán et al. developed an injectable piezoelectric hydrogel (PiezoGEL) by incorporating piezoelectric barium titanate nanoparticles into a gelatin methacryloyl (GelMA) hydrogel matrix, enabling continuous generation of localized electrical cues in response to physiological mechanical stimuli such as mastication. The hydrogel not only achieved stable filling of periodontal defects but also harnessed mechanically induced piezoelectric effects to exert sustained bioactivity, thereby enhancing antibacterial efficacy, modulating the inflammatory microenvironment, and further promoting alveolar bone regeneration. Compared with conventional static hydrogels, this mechanoresponsive strategy more closely recapitulates the dynamic oral environment and provides a promising design paradigm for simultaneously improving local retention and therapeutic functionality. Nevertheless, piezoelectric hydrogels have thus far been evaluated primarily in preclinical animal models, and their long-term biosafety, mechanical durability, and clinical applicability remain to be comprehensively validated (Roldan et al., 2023).

### 2.3 Immune-inflammatory barrier

#### 2.3.1 Immune-inflammatory microenvironment

Although LDDSs can overcome the anatomical constraints of the periodontal pocket and persistent GCF-mediated clearance to achieve prolonged local retention, the subsequent intratissue distribution, release behavior, and therapeutic efficacy of the delivered agents remain influenced by the immune-inflammatory microenvironment (Sima et al., 2019). Unlike physical barriers such as the biofilm matrix and gingival tissues, the immune-inflammatory microenvironment represents a dynamic biological barrier to local drug delivery. Rather than simply impeding drug transport through structural obstruction, this barrier compromises delivery efficiency through multiple biological processes, including immune cell-mediated phagocytic clearance, the persistent production of inflammatory mediators, and material degradation driven by oxidative stress and proteolytic enzymes (Usui et al., 2021).

During the progression of periodontitis, persistent stimulation by the dysbiotic biofilm drives the continuous recruitment of immune cells, including macrophages, neutrophils, and T lymphocytes, to periodontal lesions. Among these, macrophages play a dual role as central regulators of the inflammatory response and key determinants of the fate of nanocarriers following local administration. Because most nanoscale and microscale delivery systems are recognized as exogenous particulate materials, they are readily internalized by macrophages after entering inflamed periodontal tissues. This phagocytic clearance reduces the local accumulation of therapeutics at the target site and shortens their duration of action, thereby compromising overall drug delivery efficiency (Mo et al., 2024). Pan et al. systematically summarized the mechanisms underlying macrophage-mediated clearance of nanocarriers within inflammatory microenvironments, highlighting that particle size, surface charge, and surface functionalization all markedly influence phagocytic uptake. Accordingly, rational engineering of nanoparticle surface properties can partially reduce nonspecific macrophage uptake and improve the retention of therapeutics at the diseased site. Nevertheless, these strategies primarily enhance the pharmacokinetic behavior and local retention of delivery systems rather than fundamentally correcting the persistent immune dysregulation characteristic of periodontitis. Consequently, their long-term therapeutic efficacy remains constrained by the unresolved inflammatory microenvironment (Pan W. et al., 2021).

Beyond immune cell-mediated clearance, persistent inflammation further compromises the stability of LDDSs through the excessive accumulation of pro-inflammatory mediators and reactive oxygen species (ROS). Elevated ROS levels not only directly oxidize therapeutic agents but also induce polymer degradation and structural disruption of delivery vehicles, thereby accelerating premature drug release and reducing the amount of therapeutics reaching the target tissue. In parallel, the sustained overexpression of matrix metalloproteinases (MMPs) and other proteolytic enzymes within periodontal lesions continuously degrades natural polymer-based biomaterials, further shortening their local retention. Collectively, these inflammation-driven processes make it difficult for conventional LDDSs to maintain controlled drug release and durable therapeutic efficacy, even after successful delivery to the diseased site (Gan et al., 2023).

#### 2.3.2 Strategies for modulating the immune-inflammatory microenvironment

To overcome the biological barriers imposed by the immune and inflammatory microenvironment in periodontitis, the design paradigm of LDDSs has evolved from simply enhancing carrier stability toward actively remodeling the local immune microenvironment. Current strategies primarily focus on three complementary aspects: minimizing immune recognition and phagocytic clearance, maintaining redox homeostasis, and reprogramming immune cell functions. Collectively, these approaches aim to improve the stability and therapeutic persistence of delivery systems within inflamed periodontal tissues while promoting functional periodontal tissue regeneration.

Minimizing immune recognition and clearance of delivery vehicles represents a primary strategy for improving the efficiency of local drug delivery. Upon entering inflamed periodontal tissues, nanocarriers readily adsorb proteins derived from plasma or GCF, leading to the formation of a protein corona that facilitates macrophage recognition and phagocytic uptake, thereby resulting in premature therapeutic clearance. To mitigate this process, a variety of surface engineering strategies have been developed, including polyethylene glycol (PEG) modification, zwitterionic polymer coatings, and cell membrane biomimetic camouflage. These approaches generate a highly hydrated interfacial layer on the carrier surface, thereby minimizing opsonin adsorption and protein corona formation, reducing macrophage uptake, and ultimately prolonging the local retention of therapeutics at the diseased site (Jin et al., 2018). Importantly, the goal of these strategies is not to abolish immune recognition but to achieve a balanced interaction between delivery systems and the host immune system by minimizing nonspecific clearance while preserving biocompatibility. Such immune-evasive designs prolong carrier persistence within inflamed tissues, thereby facilitating sustained drug release and improving overall therapeutic efficacy.

Beyond minimizing immune clearance, maintaining local redox homeostasis has emerged as another major strategy for immunomodulation in periodontal drug delivery. Conventional antioxidant approaches primarily aim to eliminate excessive reactive oxygen species (ROS); however, accumulating evidence indicates that ROS not only mediate oxidative damage but also function as essential signaling molecules involved in immune regulation, cell migration, and tissue regeneration. As a result, next-generation LDDSs have shifted their focus from indiscriminate ROS scavenging toward the dynamic maintenance of redox homeostasis. As a representative example, Gan et al. developed a ROS-responsive injectable hydrogel incorporating boronate ester linkages that selectively responded to the ROS-enriched periodontal microenvironment, enabling on-demand drug release while simultaneously attenuating excessive ROS levels. The system was further combined with a complement C5a receptor antagonist and a mechanoregulatory strategy, thereby restoring macrophage phagocytic function, alleviating persistent inflammation and alveolar bone loss, and demonstrating the therapeutic potential of integrating redox regulation with immune remodeling. Nevertheless, this platform has thus far been validated primarily in a rat model of periodontitis, and its long-term stability and therapeutic performance in the complex inflammatory microenvironment of human periodontitis remain to be further investigated (Gan et al., 2023).

Furthermore, strategies that solely focus on enhancing carrier stability or modulating reactive oxygen species (ROS) levels remain insufficient to fully restore immune homeostasis within periodontal tissues. Collectively, active reprogramming of immune cell functions has emerged as an important direction in the design of next-generation LDDSs. Current approaches primarily achieve immunomodulation through the delivery of small molecules, nucleic acids, or bioactive factors to regulate macrophage polarization and associated signaling pathways, including NF-κB, Nrf2, and MAPK. These interventions promote the phenotypic transition of macrophages from a pro-inflammatory M1 state toward an anti-inflammatory, pro-regenerative M2 phenotype, thereby downregulating the expression of pro-inflammatory cytokines such as TNF-α and IL-1β while enhancing the secretion of anti-inflammatory mediators including IL-10 and TGF-β, ultimately creating a microenvironment conducive to periodontal tissue regeneration (González-Quintanilla et al., 2026).

As a representative example, Xu et al. developed a microenvironment-responsive metal–polyphenol nanozyme-based delivery platform, in which the nanozyme exhibited intrinsic superoxide dismutase (SOD)- and catalase (CAT)-like activities to continuously scavenge excessive ROS. In addition, the system simultaneously activated Nrf2/NF-κB signaling pathways, thereby promoting macrophage polarization toward the M2 phenotype and upregulating osteogenesis-related gene expression. *In vivo* studies demonstrated that this platform enabled simultaneous antibacterial, anti-inflammatory, and bone-regenerative effects, highlighting immune reprogramming as a key strategy for improving the long-term therapeutic efficacy of LDDSs (Xu et al., 2023).

Overall, strategies targeting the immune and inflammatory microenvironment have evolved from conventional approaches focused primarily on drug protection toward the active reconstruction of a pro-regenerative immune milieu. Through minimizing immune clearance, maintaining redox homeostasis, and modulating macrophage functions, next-generation LDDSs not only enhance therapeutic delivery efficiency at diseased sites but also contribute to the restoration of local immune homeostasis, thereby establishing a more favorable biological foundation for subsequent functional periodontal tissue regeneration.

### 2.4 Tissue regeneration barrier

#### 2.4.1 Multifactorial constraints on tissue regeneration

Following effective infection control and the gradual resolution of the aberrant immune-inflammatory response, the therapeutic focus of periodontal treatment shifts from halting disease progression toward the reconstruction of a functional periodontal attachment apparatus. Unlike conventional bone defect repair, periodontal regeneration requires the coordinated restoration of multiple specialized tissues, including alveolar bone, cementum, and the periodontal ligament (PDL), together with the well-organized insertion of Sharpey’s fibers, so as to re-establish the biomechanical support and biological functionality of the periodontium. However, periodontal tissue regeneration is inherently constrained by multiple interrelated factors, including insufficient angiogenesis, limited stem cell recruitment, competition for regenerative space, and a persistently dysregulated microenvironment, rather than being solely attributed to inadequate osteogenic capacity (de Souza Araújo et al., 2024; Liu et al., 2024).

First, insufficient angiogenesis represents a critical limitation for periodontal tissue regeneration by restricting both nutrient supply and recruitment of reparative cells. Persistent chronic inflammation disrupts the local microvascular network within periodontal defects, leading to sustained hypoxia and impaired perfusion. This, in turn, compromises the transport of oxygen, nutrients, and growth factors, while simultaneously attenuating the migration, survival, and osteogenic differentiation capacity of periodontal ligament stem cells (PDLSCs) and other mesenchymal stem cells. Accumulating evidence suggests that enhancing local vascularization not only improves tissue oxygenation but also facilitates stem cell recruitment and promotes the maturation of newly formed tissues, thereby serving as a fundamental prerequisite for stable periodontal regeneration (Liu et al., 2019; Sun et al., 2023).

Secondly, periodontal regeneration is not merely defined by new bone formation, but by the restoration of the hierarchical bone–periodontal ligament–cementum (bone–PDL–cementum) complex. Cementum serves as the essential anchoring interface for Sharpey’s fiber insertion onto the root surface, whereas the highly organized alignment of periodontal ligament (PDL) fibers determines the biomechanical load transmission function of the periodontium. Therefore, even in cases where substantial new bone formation is achieved, the absence of cementum regeneration or the lack of oriented PDL fiber insertion may still prevent the restoration of true functional periodontal attachment. Araújo et al. developed a three-dimensional bioprinted scaffold using PDLSC-laden collagen bioinks, in which periodontal ligament stem cells (PDLSCs) exhibited aligned spatial organization along the scaffold architecture and contributed to the formation of PDL-like tissue. These findings highlight that spatial tissue organization plays a decisive role in achieving functional periodontal regeneration (de Souza Araújo et al., 2024).

Meanwhile, according to the principle of guided tissue regeneration (GTR), epithelial cells and gingival fibroblasts exhibit significantly faster migration rates compared with periodontal ligament cells and osteogenic cells. As a result, during the early stages of wound healing, these rapidly proliferating cells preferentially colonize the root surface and defect area, leading to the formation of a long junctional epithelium or fibrous repair tissue, thereby preventing cementum regeneration and periodontal ligament formation (Mali et al., 2011). To address this issue, Liang et al. designed a three-layer electrospun membrane with a hierarchical structure, in which different regions were separately functionalized with extracellular matrix (ECM) components tailored for periodontal ligament stem cells (PDLSCs) and bone marrow-derived mesenchymal stem cells, respectively. This spatially compartmentalized design enables the simultaneous regeneration of soft and hard periodontal tissues while effectively promoting the reconstruction of the spatial architecture of the periodontal complex. These findings highlight that rational spatial organization of biomaterials plays a critical role in overcoming competitive healing processes and achieving functional periodontal regeneration (Liang et al., 2024).

Furthermore, even after the initial resolution of inflammation, a residual pathological microenvironment may persist within periodontal tissues, characterized by sustained oxidative stress, elevated expression of matrix metalloproteinases (MMPs), and aberrant release of inflammatory mediators. These adverse conditions not only impair the survival and osteogenic/cementogenic differentiation of stem cells but also disrupt collagen fiber maturation and the re-establishment of functional periodontal attachment (Zhao et al., 2023). Kiyota et al. demonstrated in a canine three-wall intrabony defect model that the promotion of cementogenesis can simultaneously induce coordinated regeneration of cementum, periodontal ligament (PDL), and alveolar bone. These findings further support the concept that functional periodontal regeneration should be established on the basis of cementum formation and the coordinated reconstruction of periodontal tissues, rather than being solely dependent on an increase in bone formation (Kiyota et al., 2024).

#### 2.4.2 Strategies for promoting periodontal tissue regeneration

In response to multiple limitations in periodontal tissue regeneration, including insufficient vascularization, restricted recruitment of reparative cells, difficulties in establishing functional periodontal attachment, and the persistence of a dysregulated microenvironment, LDDSs have evolved from simple carriers for osteogenic factor delivery into multifunctional local therapeutic platforms capable of regulating multiple key regenerative processes. Importantly, the ultimate goal of periodontal regeneration is not merely the formation of new bone, but the coordinated reconstruction of alveolar bone, cementum, and periodontal ligament, thereby restoring a structurally and functionally competent periodontal attachment apparatus with appropriate biomechanical properties (El-Nablaway et al., 2024). Accordingly, the design paradigm of LDDSs has progressively evolved from single-tissue regenerative strategies toward the coordinated regulation of multiple interdependent regenerative processes, aiming to achieve the holistic reconstruction of a functional periodontal attachment apparatus. These biological events are highly interdependent and dynamically regulated, and their precise spatiotemporal coordination is a critical determinant of both the quality and long-term stability of periodontal regeneration.

Restoration of the local vascular network is generally considered the initiating event in periodontal tissue regeneration. Chronic inflammation leads to sustained disruption of the microvascular architecture within periodontal defects, resulting in persistent hypoxia and insufficient nutrient supply, which in turn compromises the survival of reparative cells and subsequent tissue reconstruction. Accordingly, current LDDS strategies commonly aim to improve local microcirculation through the sustained delivery of pro-angiogenic factors or the development of biomaterials with intrinsic angiogenic bioactivity. Notably, recent advances in original research have further redefined the role of angiogenesis in periodontal regeneration. Beyond its traditional function in oxygen and nutrient delivery, angiogenesis is now recognized as a critical process for establishing a vascular niche that orchestrates the interactions among endothelial cells, osteogenic cells, and progenitor cell populations. This concept has been further validated by recent experimental evidence. Liu et al. employed single-cell RNA sequencing combined with *in vivo* periodontal bone defect models and demonstrated that PDGFRA^+^ dental follicle stem cells and CD31^+^EMCN^+^ endothelial cells form a functional vascular niche through VEGFA–PDGFBB signaling crosstalk. Within regenerated bone, osteogenic cells were found to be closely associated with these vascular structures, resulting in significantly enhanced defect repair compared with controls. These findings directly indicate that the fundamental role of angiogenesis in periodontal regeneration lies in the establishment of an osteogenesis-supportive vascular–bone coupled niche, rather than merely increasing vessel density (Liu et al., 2025a).

On the basis of restored local vascularization, enhancing the recruitment efficiency of endogenous reparative cells has emerged as a critical strategy for further improving periodontal regenerative potential. Currently, sustained local delivery of the chemokine stromal cell-derived factor-1 (SDF-1/CXCL12) to establish a stable chemotactic gradient represents a widely used approach to promote mesenchymal stem cell homing. Liang et al. demonstrated in a rat periodontal bone defect model that local administration of SDF-1 significantly recruited CXCR4^+^ cells and CD90^+^/CD34^-^ stromal cell populations to the defect site. Moreover, combined treatment with SDF-1 and Exendin-4 resulted in significantly greater new bone volume and trabecular thickness compared with either SDF-1 or Exendin-4 alone. These findings further indicate that reparative cell recruitment not only determines the local cellular abundance within the defect area but also governs subsequent tissue remodeling through sustained cellular replenishment. Therefore, endogenous cell homing should be regarded as an upstream regulatory event in periodontal regeneration rather than an independent therapeutic endpoint. Enhancing the efficiency of reparative cell recruitment is thus fundamentally important for ensuring a continuous supply of regenerative cells, rather than merely increasing local cell numbers (Liang et al., 2021).

In recent years, the conceptual understanding of periodontal tissue regeneration has undergone a significant shift. An increasing body of evidence suggests that periodontal regeneration should not be equated with simple bone regeneration, but rather with the coordinated reconstruction of alveolar bone, cementum, and periodontal ligament (PDL), ultimately restoring a functionally competent periodontal attachment apparatus with appropriate biomechanical properties. Based on this evolving concept, LDDS strategies have increasingly focused on the spatial reconstruction and functional restoration of periodontal ligament tissues. For instance, a study utilizing a three-dimensional bioprinted collagen scaffold loaded with periodontal ligament stem cells (PDLSCs) demonstrated that regulating cellular spatial organization and oriented growth can promote the formation of PDL-like tissue and enhance integration at the root surface. More importantly, this work further emphasizes the critical role of spatial organization within the periodontal ligament in achieving functional attachment regeneration, suggesting that true periodontal regeneration should aim at restoring the ordered alignment of PDL fibers and the continuity of the tooth–tissue interface, rather than merely increasing mineralized tissue formation (de Souza Araújo et al., 2024).

Consistent with this concept, an increasing number of studies have shifted their focus from isolated bone formation toward the integrated reconstruction of the cementum–periodontal ligament–alveolar bone interface. For instance, an extracellular matrix-mimetic strontium-doped nanofibrous microsphere delivery platform has been developed to simultaneously regulate cell adhesion, angiogenesis, osteogenic differentiation, and material degradation processes, thereby achieving more complete periodontal interfacial regeneration. Notably, this study highlights that the dynamic coupling between material degradation and tissue regeneration is critical for the formation of a functionally competent periodontal attachment. This further suggests that successful periodontal regeneration does not rely on the formation of a single tissue type, but rather on the synchronized reconstruction of the alveolar bone, cementum, and periodontal ligament interfaces during the regenerative process (Lin et al., 2024).

Even after the achievement of initial tissue regeneration, the newly formed periodontal tissues may remain susceptible to persistent low-grade inflammation, residual anaerobic bacterial colonization, oxidative stress, and immune imbalance, all of which can impair tissue maturation and even trigger secondary breakdown. Accordingly, LDDS strategies have increasingly evolved from the sole delivery of pro-regenerative cues toward the long-term maintenance of a favorable regenerative microenvironment, aiming to simultaneously regulate infection control, oxygen availability, and immune homeostasis to establish a more stable local milieu for tissue repair. A representative study developed an oxygen-releasing thermosensitive hydrogel loaded with bone marrow mesenchymal stem cell-derived small extracellular vesicles (sEVs). The results demonstrated that, compared with sEVs alone, this system not only alleviated local hypoxia and suppressed anaerobic bacterial growth but also significantly enhanced alveolar bone regeneration (Ming et al., 2024). Similarly, another study employing a multifunctional injectable hydrogel loaded with stem cells from human exfoliated deciduous teeth (SHED)-derived exosomes demonstrated that sustained modulation of the local immune microenvironment and induction of macrophage polarization toward an M2 reparative phenotype can further promote periodontal tissue maturation and long-term stable regeneration. Collectively, these findings indicate that the maintenance of a pro-regenerative microenvironment should be regarded as an integral component of periodontal tissue regeneration, rather than a purely supportive strategy (Yu et al., 2024). These original studies further indicate that the regenerative microenvironment is not merely a background condition for periodontal tissue repair, but a critical determinant that governs the sustained functionality of regenerative signaling. Accordingly, LDDS design has shifted from simple delivery of regenerative factors toward construction of a pro-regenerative niche that actively supports tissue repair.

Taken together, recent original studies suggest that the development of LDDSs has shifted from improving single regenerative outcomes toward coordinating the spatiotemporal relationships among multiple key biological events. However, most current strategies still focus on optimizing individual processes, such as angiogenesis, cell recruitment, or tissue reconstruction, in a relatively isolated manner, with limited integration or programmed coordination among different regenerative phases. Therefore, achieving precise spatiotemporal coupling and orchestrated regulation of multistage biological events has emerged as a central challenge and a key direction for the next-generation of periodontal LDDSs.

## 3 LDDSs: from single-targeting to multilevel synergy

**FIGURE 3:**
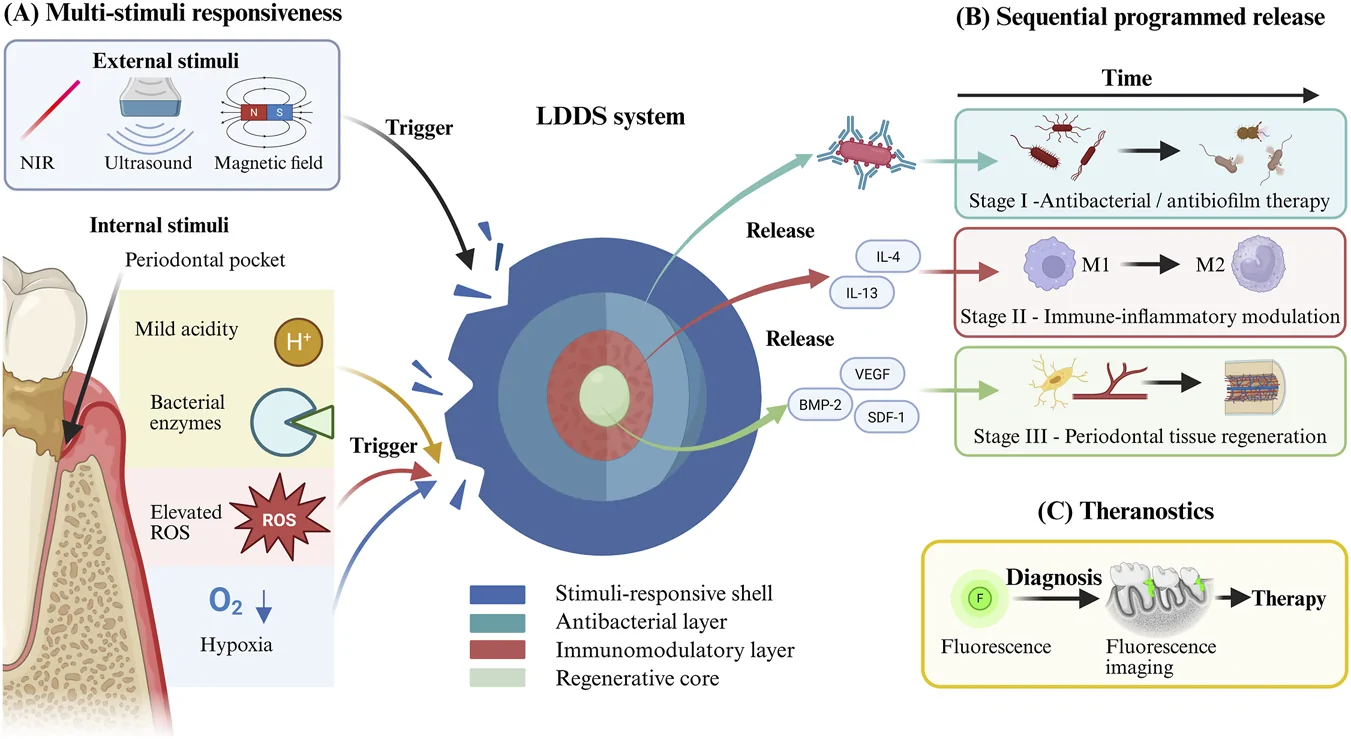
Representative design directions of advanced periodontal LDDSs. Recent studies on periodontal LDDSs have mainly advanced along three related but distinct directions: multi-stimuli-responsive delivery, sequential programmed release, and theranostic integration. **(A)** Multi-stimuli-responsive systems exploit external triggers, such as near-infrared light, ultrasound, and magnetic fields, or endogenous periodontal cues, including mild acidity, bacterial enzymes, elevated ROS, and hypoxia, to enable microenvironment-adaptive activation. **(B)** Sequential programmed-release systems aim to match the antibacterial–anti-inflammatory–regenerative sequence of periodontal therapy. **(C)** Theranostic LDDSs further incorporate diagnostic or imaging-guided functions to support feedback-informed intervention.

As the complexity and temporal dynamics of the periodontal microenvironment have become increasingly recognized, LDDS design has moved beyond single-function or single-stimulus-responsive carriers. Recent representative studies discussed in this section highlight three major and complementary design directions: multi-stimuli-responsive systems for microenvironment-adaptive activation, sequential programmed-release systems for stage-specific antibacterial–anti-inflammatory–regenerative intervention, and theranostic systems for imaging-guided diagnosis and treatment. Figure 3 summarizes these emerging directions and their shared goal of improving the spatiotemporal precision of periodontal local therapy.

### 3.1 Design of multi-stimuli-responsive systems

As our understanding of the periodontal microenvironment deepens, LDDS design has evolved from single-stimulus responsiveness toward multidimensional synergistic targeting. Early systems predominantly relied on individual endogenous triggers (e.g., pH or enzymes). However, biochemical cues within periodontal lesions exhibit pronounced dynamic fluctuations and spatial heterogeneity (Chen J. et al., 2025). Therefore, single-stimulus-responsive mechanisms often fail to achieve consistent and precise drug release. For instance, local acidity in mildly inflamed regions may be insufficient to activate pH-responsive carriers, whereas transient and nonspecific oxidative stress may induce premature drug release in ROS-responsive systems. Both scenarios disrupt the spatiotemporal control of drug release, reducing overall therapeutic efficacy (Hu et al., 2024; Qi, Wang, Wang, Xie and Chen, 2024). To address these limitations, delivery strategies integrating multiple pathological cues have been developed. These systems typically require the simultaneous presence of two or more stimuli to initiate drug release, thereby improving targeting specificity and enhancing system stability (Wu et al., 2022).

To address the complexity of the periodontal microenvironment, a variety of dual- and multi-stimuli-responsive systems based on combined endogenous stimuli have been engineered. Among these, pH/enzyme dual-responsive systems are among the most extensively studied. In such systems, the mildly acidic environment generated by bacterial metabolism induces carrier protonation, swelling, or charge reversal, promoting exposure of internal responsive domains. Subsequently, enzymes such as MMPs or bacterial proteases cleave specific chemical linkages, triggering localized drug release at infected sites (Jian et al., 2026; Wang et al., 2022; Yin et al., 2024). Similarly, ROS/temperature dual-responsive systems have also been widely explored. These platforms commonly employ thermosensitive hydrogels that undergo *in situ* gelation at physiological temperature, enhancing local retention (Wang et al., 2026). Concurrently, incorporation of ROS-sensitive linkages (e.g., thioketals or boronate esters) renders release kinetics responsive to oxidative stress levels, enabling adaptive drug delivery that correlates with the inflammatory severity (Li et al., 2024; Liu et al., 2022; Zhu et al., 2025). Furthermore, advanced combinatorial systems, such as hypoxia/ROS or hypoxia/mild acidity dual-responsive platforms, have been developed to target the severely compromised microenvironment of deep periodontal pockets. These systems specifically address hypoxic niches where anaerobic bacteria predominate, reducing nonspecific drug leakage in superficial tissues (Chen et al., 2023). Beyond these conventional combinations, novel endogenous signal-responsive systems continue to emerge. For example, pH-responsive hydrogels derived from the plant-based molecule embelin regulate drug release through dynamic chemical bond rearrangement. Under acidic conditions, these systems not only exert intrinsic antibacterial activity but also modulate macrophage polarization and suppress pro-inflammatory cytokine expression, improving the local immune microenvironment (Cai et al., 2024). Collectively, multi-endogenous signal-driven systems enhance the alignment between drug release behavior and dynamic microenvironmental changes. This alignment facilitates a coordinated therapeutic sequence across antibacterial activity, immunomodulation, and tissue regeneration.

Precise control over drug release across different treatment stages remains challenging when response systems rely solely on endogenous pathological signals. For example, rapid, high-dose release is typically required during the acute phase of periodontal infection, whereas sustained, low-dose delivery is preferable during the tissue regeneration phase (Kamaly et al., 2016). Consequently, synergistic strategies integrating exogenous stimuli with endogenous signals have attracted increasing attention (Li F. et al., 2020). Common exogenous stimuli include near-infrared (NIR) irradiation, ultrasound, and alternating magnetic fields. By coupling these physical stimuli with endogenous factors, such as pH and enzyme activity, dual- or multi-stimuli-responsive systems can be engineered to achieve more precise regulation of drug release. Among various exogenous response strategies, PDT and photothermal therapy (PTT) have been widely employed (Huang et al., 2023; Wang et al., 2025; Wen et al., 2026). In NIR-responsive systems, for example, external irradiation can directly disrupt bacterial structures through photothermal effects while enhancing biofilm permeability. Furthermore, this process can be further integrated with nanozyme-catalyzed reactions to modulate local oxidative stress. Additionally, the incorporation of nitric oxide (NO) donors enables controlled NO release under photothermal stimulation, thereby further inhibiting bacterial activity and disrupting biofilm stability. Concurrently, NO is also involved in local immunomodulation (Li Z. et al., 2025). By integrating multiple mechanisms, including photothermal effects, gas release, and oxidative stress regulation, into a single platform, synergistic antibacterial and immunomodulatory effects can be achieved, thereby improving the overall efficacy of periodontal therapy (Li Z. et al., 2025). Collectively, these advances have expanded multi-stimuli-responsive LDDSs from conventional biochemical responsiveness toward increasingly sophisticated and precisely controllable platforms.

Beyond these established approaches, recent advances have further extended multi-responsive strategies to externally controllable and mechanically activated platforms. These emerging approaches enable more precise spatiotemporal regulation of therapeutic activation and drug release. Ultrasound-responsive systems have attracted increasing attention because ultrasound can noninvasively penetrate deep tissues and remotely activate therapeutic agents or prodrugs with high spatial selectivity (Fu and Hu, 2024). Piezoelectric biomaterials have also emerged as promising delivery platforms by converting endogenous biomechanical forces or externally applied mechanical stimulation into localized electrical signals capable of regulating drug release while simultaneously promoting tissue regeneration (Li A. et al., 2024). Mechanophore-based polymers further extend this concept by activating therapeutic molecules through force-induced chemical bond cleavage, providing a new strategy for mechanically programmable drug delivery (Shi Y. et al., 2023). Although these technologies remain largely at the proof-of-concept stage and have rarely been explored in periodontal LDDSs, they substantially broaden the design paradigm of multi-stimuli-responsive systems and may provide new opportunities for on-demand and spatiotemporally precise periodontal drug delivery (Fu and Hu, 2024; Li A. et al., 2024; Shi Z. et al., 2023).

### 3.2 Programmed drug release aligned with the antibacterial–anti-inflammatory–regenerative sequence

Although multi-stimuli-responsive systems improve the spatial selectivity of drug release, they are primarily designed to respond to local pathological stimuli in a trigger-dependent manner. In contrast, periodontal tissue repair proceeds through distinct phases, typically comprising infection control, immunomodulation, and tissue regeneration. Each stage requires specific therapeutic agents and distinct release kinetics. Traditional local delivery systems, however, predominantly employ a synchronous drug release profile, which fails to accommodate these temporal transitions. For example, during the early stage, before periodontal infection is resolved, premature release of osteogenic factors often leads to their degradation and inactivation within the inflammatory microenvironment. Conversely, during the later stages of repair, prolonged exposure to high concentrations of antimicrobials may impair cellular function, thereby hindering periodontal tissue regeneration (Algazlan et al., 2022; Chen and Jin, 2010). Collectively, the development of programmed release systems tailored to the antibacterial–anti-inflammatory–regenerative sequence has emerged as a primary focus in current LDDS design.

From a materials design perspective, programmed release is typically achieved through the construction of hierarchical structures, such as microsphere–hydrogel composites or layered scaffolds. These systems incorporate distinct drug reservoirs to enable the sequential release of multiple functional molecules (Khademi et al., 2025). Typically, antimicrobial agents are first released from the outer layers or rapidly degrading components to reduce the initial bacterial load (Cai et al., 2024). Subsequently, as the bulk material degrades, anti-inflammatory or osteogenic factors are gradually released from the internal compartments, thereby promoting periodontal tissue repair during later stages (Chang et al., 2013). Furthermore, delayed release of osteogenic or angiogenic factors is typically achieved by encapsulation within microspheres or highly cross-linked networks (Mu et al., 2025).

Beyond passive release mechanisms driven by material degradation, actively responsive systems tailored to microenvironmental cues have gained increasing attention. These systems enable dynamic regulation by coupling drug release to local pH, enzyme activity, and oxidative stress (Cai et al., 2024; Li W. et al., 2025). This cascade release mechanism, driven by dynamic microenvironmental cues, enhances the synchronization between drug delivery and disease progression.

Despite these advances in microenvironment-responsive drug release, most programmed release systems are still developed and evaluated under relatively static experimental conditions. However, the oral cavity represents a highly dynamic environment characterized by continuous salivary flow, mastication, speech, and mechanical stress, all of which may compromise material retention and structural stability, thereby posing substantial challenges to maintaining the intended spatiotemporal drug delivery profile of LDDSs (Liu et al., 2025c). To better address these challenges, recent research has increasingly emphasized environment-adaptive material design to improve LDDS performance under clinically relevant oral conditions (Liu et al., 2025c). For example, fatigue-resistant injectable hydrogels have been developed to preserve structural integrity under repeated mechanical loading (Han et al., 2025), while sprayable rapid-gelling mucoadhesive systems (Liu et al., 2025b) and reservoir-based local delivery platforms (Alghanem et al., 2025) have demonstrated prolonged local retention despite continuous oral movement, thereby facilitating sustained local drug delivery under dynamic oral conditions. These results indicate that future programmed LDDSs should integrate pathological microenvironment-responsive drug release with mechanically adaptive material design to achieve more reliable spatiotemporal drug delivery under dynamic oral conditions.

Building upon these principles, programmed delivery strategies have evolved toward achieving multidimensional pharmacological synergy. Given the temporal overlap of the anti-inflammatory, angiogenic, and osteogenic phases during periodontal tissue repair (Li X. L. et al., 2025), multiple bioactive inorganic ions (e.g., Sr^2+^, Zn^2+^, Cu^2+^) have been incorporated into these systems. Upon initial release, these ions exert antibacterial effects by disrupting bacterial membranes or inducing oxidative stress; conversely, their sustained low-dose release during the intermediate and late stages modulates the immune microenvironment and activates osteogenic signaling pathways, thereby promoting bone formation and tissue regeneration (Huang et al., 2025; Tang et al., 2025; Yu et al., 2026). Incorporation of such bioactive components simplifies system architecture while maintaining continuous biological regulation.

### 3.3 Achieving precise control of LDDSs via theranostic integration

In conventional periodontitis treatment, clinical assessment relies predominantly on periodontal probing, which fails to accurately reflect bacterial load and microenvironmental conditions within deep periodontal pockets. Although programmed release strategies have improved synchronization with disease progression, their regulation remains fundamentally dependent on predefined responsive mechanisms. Therefore, these systems lack real-time feedback, thereby limiting dynamic adjustment of therapeutic strategies based on *in situ* lesion conditions. To overcome this limitation, LDDS design is increasingly shifting toward integrated theranostic platforms. Among available imaging modalities, photoacoustic imaging (PAI) and near-infrared II (NIR-II) fluorescence imaging are widely used for periodontal lesion visualization (Lin et al., 2025; Qin et al., 2026). PAI combines optical contrast with ultrasonic resolution to enable deep-tissue imaging, whereas NIR-II fluorescence imaging provides low background noise and high tissue penetration, thereby enhancing detection sensitivity (Qin et al., 2026).

In the design of diagnostic modules, probe targeting capability and responsiveness are of paramount importance. For example, fluorescent dyes can be conjugated to specific peptide sequences to construct enzyme-responsive probes targeting periodontal pathogens; these probes recover fluorescence upon enzymatic cleavage, thereby enabling detection of infected regions (Moore et al., 2022). Another strategy involves functionalizing rare-earth-doped upconversion nanoparticles with the bacteria-targeting peptide UBI29–41, enabling specific adsorption onto phosphatidylglycerol on bacterial membranes via electrostatic interactions (Lin et al., 2025). Upon encapsulation within a zeolitic imidazolate framework (ZIF-8), the nanoparticles remain in a fluorescence-quenched state under physiological conditions. Upon exposure to an acidic inflammatory microenvironment, proton-mediated dissociation of the ZIF-8 framework triggers the release of encapsulated components, thereby rapidly restoring the NIR-IIb fluorescence signal and enhancing imaging specificity (Lin et al., 2025).

Following acquisition of spatial information and pathological feedback from the lesion, targeted therapy can be initiated through external light irradiation. For example, liposome-encapsulated aggregation-induced emission (AIE) molecules can generate a robust photothermal effect under near-infrared irradiation, thereby inhibiting bacterial growth. Photothermal therapy induces localized hyperthermia to disrupt bacterial structures, exhibiting broad-spectrum bactericidal activity against periodontal pathogens (Zhang et al., 2025). Furthermore, PDT is commonly integrated into these theranostic platforms. By incorporating photosensitizers (e.g., chlorin e6, Ce6), light energy is converted into highly cytotoxic singlet oxygen under 660 nm irradiation, thereby enabling efficient eradication of periodontal biofilms.

To overcome the limitations of PDT in hypoxic deep periodontal pockets and mitigate inflammation-induced tissue damage, nanomaterials with enzyme-mimicking activity (nanozymes) are often incorporated. Molybdenum- or manganese-based nanozymes, characterized by abundant oxygen vacancies, exhibit intrinsic catalase- and superoxide dismutase-like activities. These nanozymes catalyze decomposition of excess hydrogen peroxide into oxygen, thereby alleviating hypoxia, suppressing anaerobic bacterial overgrowth, and replenishing oxygen required for PDT (Lin et al., 2025). Moreover, these platforms often incorporate stimuli-responsive matrices (e.g., MMP-responsive hydrogels) that degrade within the inflammatory microenvironment to release bioactive metal ions or anti-inflammatory agents (e.g., paeonol) for local immunomodulation (Qin et al., 2026). These bioactive components not only promote angiogenesis and osteogenesis but also regulate macrophage polarization, thereby accelerating inflammation resolution and tissue repair (Lin et al., 2025). By integrating diagnostic imaging, antimicrobial therapy, and microenvironmental regulation, these advanced systems enable dynamic control of periodontitis treatment, offering new paradigms for precise intervention in complex lesions (Lin et al., 2025; Qin et al., 2026).

## 4 Clinical translation bottlenecks of LDDSs

**FIGURE 4:**
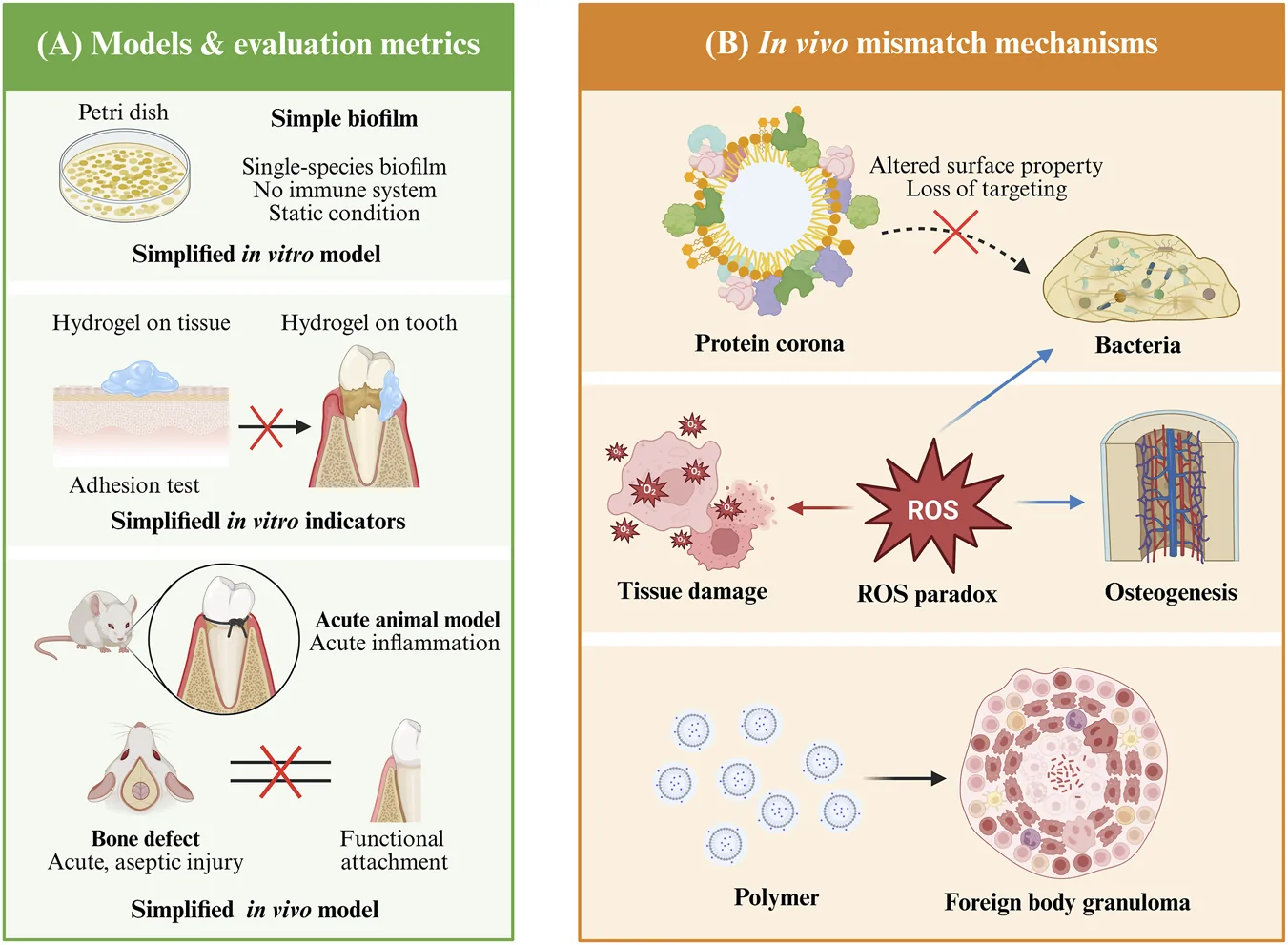
Barriers to clinical translation of LDDSs in periodontitis. **(A)** Representative models and *in vitro* indicators, including biofilm models, adhesion tests and animal models. **(B)** Major mechanisms contributing to *in vitro*–*in vivo* mismatch, including protein corona formation, ROS paradox and foreign body responses.

Despite continuous advances in LDDS materials design, successful clinical translation remains limited. This translational gap arises from multiple factors, including discrepancies between experimental models and the clinical periodontal microenvironment, as well as challenges related to *in vivo* stability, drug release behavior, and safety (Figure 4).

To facilitate comparison, representative LDDSs associated with the major translational barriers discussed in this section are summarized in Table 1, together with their preclinical strengths, clinical translation challenges, and corresponding design implications.

**TABLE 1:** Preclinical strengths and clinical translation challenges of representative periodontal LDDSs and regenerative platforms.

Translational challenge	Representative study	Preclinical strengths	Clinical translation challenge	Design implication
Simplified biofilm models	Prado et al. (2020) — multispecies periodontal biofilm model	Established a dynamic multispecies periodontal biofilm model using tooth-root substrates and stirred bioreactor culture; biofilm formation was assessed by CFU counting, SEM, and checkerboard DNA-DNA hybridization	Improved microbial complexity but still lacks host immunity, gingival epithelium, GCF-mediated clearance, salivary flow, and mastication-related disturbance	Antibiofilm LDDSs should be tested in dynamic multispecies biofilms combined with fluid flow and periodontal pocket-like conditions
Poor IVIVC/Wet retention	Baus et al. (2019) — mucoadhesive hydrogels for buccal drug delivery	Compared rheological, tensile, ex vivo flow-retention, and in vivo residence-time assays; ex vivo artificial-saliva flow retention showed the strongest correlation with in vivo residence time	Buccal mucosa models do not reproduce deep periodontal pockets, continuous GCF outflow, subgingival biofilm, inflamed tissue, mastication, or speech-related movement	Periodontal LDDS evaluation should include simulated GCF washout, wet-tissue retention, and dynamic mechanical disturbance assays
Multifunctional hydrogel therapy	Pan P. et al. (2025) — THDB-initiated injectable hydrogel for periodontitis treatment	Developed a rapidly gelling injectable hydrogel integrating wet adhesion, fatigue resistance, antibacterial activity, ROS scavenging, osteoinduction, and rat periodontitis efficacy	Short-term animal models and simplified antibacterial assays do not fully reproduce chronic human pockets, mature biofilms, GCF clearance, enzymatic degradation, masticatory forces, or redox fluctuation	Future LDDSs should balance retention, degradation, antimicrobial activity, stage-dependent redox regulation, and regenerative support under dynamic periodontal conditions
Mandibular bone repair model	Nakano et al. (2026) — scaffold-free DPSC constructs for mandibular bone regeneration	Fabricated scaffold-free DPSC spheroid constructs by bio 3D printing; transplantation enhanced mandibular bone regeneration and donor-cell-derived osteogenic contribution	Mandibular bone repair does not fully reproduce periodontitis-associated biofilm, chronic inflammation, GCF flow, cementogenesis, PDL alignment, or functional attachment remodeling	Scaffold-free cell constructs should be further evaluated for immune compatibility, vascularization, mechanical stability, and cementum–PDL–bone interface regeneration
Stiffness-guided osteogenesis	Gao et al. (2026) — PDLSC-loaded GelMA/PEGDA scaffolds	Prepared stiffness-tunable GelMA/PEGDA scaffolds loaded with PDLSCs; increased matrix stiffness promoted osteogenic differentiation and mineralization	Controlled osteogenic models lack chronic infection, GCF washout, inflammatory cytokines, immune-cell interactions, masticatory loading, cementogenesis, and functional attachment	Regenerative LDDSs should be assessed beyond ALP, RUNX2, OCN, mineralization, and bone volume; cementogenesis and PDL organization should also be evaluated
Bioink stiffness	Zhu et al. (2023) — bioprinted PDLSCs in high-concentration GelMA hydrogels	Bioprinted PDLSCs in GelMA hydrogels with different concentrations; high-concentration GelMA enhanced osteogenic differentiation and promoted in vivo bone formation	Osteogenesis-oriented evaluation does not fully address GCF washout, multispecies biofilm, inflammatory cytokines, LPS-induced PDLSC impairment, cementum formation, or PDL alignment	Bioink stiffness should be optimized for printability, cell survival, osteogenesis, cementogenic differentiation, PDL regeneration, and cementum–PDL–bone reconstruction

These studies are not presented as confirmed clinical trial failures. They represent preclinical LDDSs, retention models, biofilm models, and regenerative platforms whose experimental performance may not fully predict clinical periodontal outcomes because GCF, washout, masticatory forces, chronic multispecies biofilms, inflammation, enzymatic degradation, redox fluctuation, and functional cementum–PDL–bone regeneration are insufficiently reproduced.

### 4.1 Inadequate recapitulation of clinical pathology by current models

Although various LDDS formulations designed for biofilm penetration and antibacterial activity demonstrate promising outcomes in experimental studies, their extrapolation to clinical applications remains highly uncertain. This primarily arises from the inability of current model systems to faithfully recapitulate the structural and functional hallmarks of *in situ* periodontal lesions.

Conventional *in vitro* antibacterial assays often rely on simplified mono- or limited multispecies biofilm models (Park et al., 2026; Prado et al., 2020). These systems lack the complex multispecies symbiosis and dynamic spatiotemporal succession characteristic of natural periodontal pockets. Consequently, they fail to reproduce the stratified architecture and stable microenvironment of mature biofilms, often leading to overestimation of antimicrobial efficacy. Additionally, static *in vitro* conditions lack dynamic interactions with biological fluids and immune components, thereby failing to capture *in vivo* alterations in material surface properties and clearance kinetics (Piotrowski-Daspit et al., 2024). This discrepancy further amplifies the gap between experimental outcomes and clinical realities.

Furthermore, *in vitro* evaluations frequently infer potential clinical efficacy solely based on physical and rheological parameters, such as storage modulus (G′), loss modulus (G″), and interfacial adhesion strength (Lee et al., 2024). Although substantial interfacial adhesion is frequently demonstrated using *ex vivo* porcine skin or oral mucosal models (Liu et al., 2023; Shao et al., 2024), some materials exhibit high adhesion strength in these models (Chen et al., 2025). However, these *ex vivo* metrics primarily reflect interfacial mechanics rather than directly correlating with clinical endpoints such as reductions in probing depth and gains in clinical attachment level. Indeed, discrepancies between *ex vivo* mucosal adhesion rankings and actual *in vivo* retention times indicate that current adhesion assays are inadequate predictors of *in vivo* performance (Baus et al., 2019).

Similar translational hurdles are also evident in animal models of periodontitis and tissue regeneration, further complicating the *in vivo* assessment of LDDS efficacy. Although acute ligation models successfully induce short-term inflammation and alveolar bone resorption (Ohsugi et al., 2022; Shi Y. et al., 2023), the resulting microbial community resembles early-stage dysbiosis, lacking the architectural complexity and immunomodulatory profiles of chronic infections (Wei et al., 2024). Therefore, therapeutic responses observed in these models cannot be directly extrapolated to chronic clinical periodontitis. Furthermore, bone defect models utilized in regeneration studies are predominantly acute, aseptic defects (Liang et al., 2025; Nakano et al., 2026). Their repair is driven by intrinsic osteogenic healing capacities, which fundamentally differ from the destructive and regenerative mechanisms underlying chronic periodontal infections.

True periodontal regeneration extends beyond mere osseous repair; it requires the synergistic reconstruction of cementum, periodontal ligament (PDL), and alveolar bone to form a functional attachment apparatus (Liu et al., 2019). Cementum serves as the critical biological interface linking alveolar bone and the periodontal ligament, providing the insertion site for Sharpey’s fibers and thereby enabling functional periodontal attachment (Zhu et al., 2025). Therefore, successful periodontal regeneration requires coordinated cementogenesis together with the re-establishment of a functional cementum–PDL–bone interface (Ozkendir et al., 2024; Zhu et al., 2025). This highlights that regenerative LDDSs should not exclusively target osteogenesis but should also support cementogenesis to facilitate functional periodontal attachment, as osteogenesis and cementogenesis require coordinated but distinct regenerative cues during periodontal healing (Ozkendir et al., 2024; Yürük et al., 2024). During this process, newly regenerated PDL fibers must be properly oriented and firmly inserted into both newly formed cementum and alveolar bone through Sharpey’s fibers to restore biomechanical stability and physiological tooth support (Park, 2019; Zhu et al., 2025). Importantly, osteoblast- and cementoblast-oriented LDDSs should not be designed or evaluated according to identical regenerative criteria (Liu et al., 2019; Park, 2019; Zhu et al., 2025). Osteoblast-targeted systems are primarily intended to enhance osteoblast differentiation and bone matrix deposition, and their efficacy is commonly evaluated by osteogenic markers such as ALP, RUNX2, and OCN expression, mineralized nodule formation, and new bone volume (Gao et al., 2026; Liang et al., 2025; Zhu et al., 2023). In contrast, cementoblast-targeted LDDSs should promote cementoblast differentiation, cementogenic matrix formation, and subsequent insertion of Sharpey’s fibers into newly formed cementum (Park, 2019; Zhu et al., 2025). Accordingly, evaluation should extend beyond bone formation to include cementogenic markers (e.g., CEMP1 and CAP), formation of acellular extrinsic fiber cementum (AEFC), and reconstruction of the functional cementum–PDL attachment apparatus (Park, 2019; Zhu et al., 2025). Therefore, simple osteogenesis observed in aseptic defect models cannot be equated with true restoration of periodontal attachment structures.

Because these studies are conducted under highly controlled and idealized conditions (Gao et al., 2026; Zhu et al., 2023), they frequently overestimate material efficacy compared with the clinical periodontal environment. In reality, deep periodontal pockets harbor a persistently infected microenvironment saturated with pathogenic bacteria, endotoxins, and proteolytic enzymes. Upon implantation of regenerative scaffolds, bacterial endotoxins, such as lipopolysaccharides (LPS), severely compromise the regenerative potential of resident stem cells. Specifically, LPS not only inhibits cellular proliferation but also disrupts multiple signaling pathways essential for periodontal regeneration. Persistent inflammatory signaling, particularly activation of the NF-κB/NLRP3 inflammasome pathway, sustains cytokine production and perpetuates the inflammatory microenvironment, thereby creating conditions unfavorable for tissue repair (Guo et al., 2024). At the same time, regenerative signaling is suppressed through inhibition of the PI3K/Akt pathway, resulting in reduced osteogenic marker expression and impaired mineralization of periodontal ligament stem cells (PDLSCs) (Cao et al., 2025). Recent studies further show that inflammatory downregulation of BMAL1 induces PANoptosis through ERK/AP-1 signaling, thereby simultaneously compromising PDLSC survival and osteogenic capacity (Wang et al., 2025). Therefore, even if a material successfully releases growth factors and recruits stem cells, persistent inflammatory signaling within the periodontal microenvironment may continue to impair stem cell survival and osteogenic differentiation, ultimately limiting functional periodontal regeneration.

### 4.2 Discrepancies between designed delivery behaviors and the *in vivo* environment

Although oxidative stress, enzymatic degradation, immune clearance, and fluid washout profoundly influence the post-implantation performance of LDDSs, the resulting translational discrepancies can be broadly categorized into three interconnected *in vivo* processes: interfacial remodeling by biological fluids, stage-dependent redox regulation, and host responses to retained biomaterials.

Although direct evidence from therapeutic periodontal LDDSs remains limited, studies using periodontitis-related saliva and antibacterial nanomaterials suggest that protein adsorption may substantially reshape the nano–bio interface of locally delivered carriers (Chen C. et al., 2026; Chen G. et al., 2026). The composition of the protein corona varies considerably among different delivery systems because the adsorption profile of proteins is largely governed by carrier surface chemistry and functional ligands (Capolla et al., 2023). The resulting protein corona confers a distinct biological identity upon LDDSs, thereby shaping their subsequent interactions with bacteria and host cells in the periodontal microenvironment (Hadjidemetriou et al., 2024). These altered nano–bio interactions may subsequently compromise the therapeutic functions originally engineered into LDDSs. Protein adsorption may therefore impair LDDS performance through multiple mechanisms. In some systems, the adsorbed protein layer acts as a physical barrier that limits effective interactions between nanomaterials and bacteria, thereby reducing antibacterial and photothermal antibacterial efficacy (Chen G. et al., 2026; Guglielmelli et al., 2023). In others, protein adsorption masks catalytic active sites and reduces nanozyme activity (Cong et al., 2024), while altered protein corona profiles may also compromise engineered targeting functions by masking surface ligands (Capolla et al., 2023). Because conventional *in vitro* models rarely account for these dynamic nano–bio interactions, the *in vivo* release kinetics, targeting efficiency, and overall therapeutic performance of LDDSs cannot be reliably predicted. To overcome these protein corona-mediated limitations, recent advances in nanomedicine are increasingly moving from viewing the protein corona as an unavoidable biological barrier to exploiting it as an engineering tool for improving LDDS performance through rational nano–bio interface design (Hadjidemetriou et al., 2024). For example, surface engineering strategies have been developed to modulate protein corona composition, thereby improving cellular uptake and drug delivery efficiency through the formation of favorable protein coronas (Miao et al., 2024). In parallel, ligand pre-adsorption strategies have been shown to preserve targeting performance despite protein corona formation by minimizing corona-induced masking of targeting ligands (Li J. et al., 2025). Although these approaches have not yet been translated into periodontal LDDSs, they provide valuable design principles for improving LDDS performance in the protein-rich periodontal microenvironment.

Second, ROS play a paradoxical role in periodontal pathogenesis and tissue repair. While immune cells generate copious amounts of ROS during inflammation to eradicate bacteria, excessive ROS production also inflicts collateral damage on surrounding periodontal tissues (Vichare et al., 2025). Accordingly, many recent delivery systems have prioritized ROS scavenging as a key design objective (Pan P. et al., 2025; Yu et al., 2026), demonstrating significant antioxidant efficacy *in vitro* (Mu et al., 2025). However, ROS are not solely deleterious. During osteogenic differentiation, they function as crucial intracellular signaling molecules; physiological fluctuations in ROS levels directly regulate osteogenic gene expression and matrix mineralization (Chen J. et al., 2025). At physiological concentrations, ROS facilitate osteoblast activation and guide immune cells to clear necrotic debris without inducing tissue damage (Wagner et al., 2024; Zhang et al., 2021). The over-scavenging of local ROS by hyperactive delivery systems can therefore inhibit essential signaling pathways, such as the ROS–APEX1–BMP2 axis (where APEX1 acts as a critical redox-sensitive mediator), thereby disrupting normal bone repair (Novak et al., 2023). Because conventional nano-antioxidant systems lack the ability to discriminate between pathological and physiological ROS, they may inadvertently suppress essential signaling functions while mitigating oxidative stress (Li X. et al., 2020). This possibility may partly explain why strong antioxidant performance *in vitro* does not always translate into effective bone regeneration *in vivo* (Shan et al., 2024).

This paradox highlights an important design principle for next-generation periodontal LDDSs. Rather than maximizing ROS scavenging, future delivery systems should dynamically regulate the local redox microenvironment in a stage-dependent manner. During the inflammatory phase, excessive ROS should be selectively attenuated to alleviate oxidative damage and suppress persistent inflammation. As inflammation resolves, antioxidant activity could be gradually reduced to permit the recovery of physiological redox signaling required for tissue repair. Recent advances in bone tissue engineering have increasingly focused on microenvironment-responsive biomaterials capable of sensing pathological cues and achieving on-demand drug release. By integrating ROS-responsive, multi-stimuli-responsive, and feedback-regulated strategies, these systems dynamically regulate the local microenvironment while promoting immunomodulation, angiogenesis, and osteogenesis (Yang et al., 2026). Representative studies have further demonstrated that ROS-responsive delivery systems can coordinate inflammation control with tissue regeneration through microenvironment-triggered drug release (Fu et al., 2025), while multifunctional ROS-responsive hydrogels further enhance immune modulation and bone regeneration (Zhang et al., 2026). These strategies may guide the rational design of future periodontal LDDSs, shifting the focus from indiscriminate ROS elimination toward more precise, dynamic, and stage-specific redox regulation.

Third, prolonging the retention time of carrier materials is a common strategy to enhance local drug concentrations and therapeutic efficacy. However, this approach—particularly when employing slow-degrading carrier systems—often provokes local foreign body reactions. In periodontal applications, polymer barrier membranes can induce the formation of foreign body granulomas that impair local healing (Chang et al., 2022). Similarly, even biodegradable materials can elicit foreign body giant cell reactions, leading to gradual encapsulation within a fibrous capsule (Thompson et al., 2025). These foreign body reactions severely hinder host–material integration, impede cellular migration into the defect site, and obstruct *de novo* tissue formation, ultimately compromising overall regenerative outcomes.

## 5 Conclusions and future perspectives

Periodontal LDDSs have evolved from conventional passive depots into multifunctional platforms capable of improving local retention, penetrating biofilms, modulating inflammation, and supporting tissue regeneration. This evolution reflects a broader shift from single-target intervention toward multilevel synergy within the periodontal microenvironment. By integrating wet-tissue adhesion, biofilm-disrupting strategies, immune regulation, programmed release, and theranostic feedback, advanced LDDSs provide new opportunities for more precise and stage-matched periodontal therapy.

Nevertheless, promising *in vitro* or preclinical performance does not necessarily predict clinical periodontal efficacy. The major translational gap lies in the discrepancy between simplified experimental settings and the dynamic, chronically inflamed, and mechanically active periodontal pocket. Current models often fail to reproduce mature multispecies biofilms, continuous GCF-mediated washout, saliva dilution, mastication- and speech-related mechanical loading, immune-cell interactions, protein-rich biological fluids, and the hierarchical cementum–PDL–bone interface. Therefore, future LDDS studies should prioritize physiologically relevant evaluation systems rather than increasing material complexity alone.

To translate these recommendations into practical experimental guidance, a do-and-don’t framework for future periodontal LDDS studies is proposed in Table 2.

**TABLE 2:** Practical do-and-don’t framework for future periodontal LDDS studies.

Do	Don’t
Use dynamic multispecies biofilm models with fluid flow and periodontal pocket-like conditions	Do not rely solely on planktonic bacteria or mono-species biofilms
Include simulated GCF washout, saliva dilution, and cyclic mechanical loading	Do not infer clinical retention only from static adhesion strength, swelling, or rheological parameters
Evaluate antibacterial, anti-inflammatory, and regenerative outcomes together	Do not use single endpoints as sufficient evidence of therapeutic efficacy
Assess cementogenesis, PDL fiber orientation, Sharpey’s fiber insertion, and functional attachment	Do not equate new bone volume with true periodontal regeneration
Test nano-LDDS behavior in saliva- or GCF-like protein-rich fluids	Do not assume targeting ligands, catalytic sites, or surface properties remain unchanged in vivo
Design stage-dependent redox regulation	Do not pursue maximal ROS scavenging throughout all healing phases
Balance material retention with timely degradation and host compatibility	Do not prolong residence time without evaluating degradation, macrophage response, and tissue integration

In parallel, material design should increasingly account for *in vivo* remodeling after administration. Protein corona formation may alter the biological identity, targeting capacity, catalytic activity, and antibacterial performance of nano-LDDSs. ROS regulation should also move beyond indiscriminate scavenging, because physiological ROS participate in osteogenic signaling and tissue repair. Future systems should therefore adopt stage-dependent redox regulation, attenuating excessive pathological ROS during inflammation while preserving physiological redox signaling during regeneration. Similarly, prolonged retention should be balanced with timely degradation and host compatibility to avoid foreign body reactions and impaired tissue integration.

Finally, true periodontal regeneration should not be evaluated solely by osteogenic markers or new bone volume. Successful regenerative LDDSs should support coordinated cementogenesis, organized PDL fiber alignment, Sharpey’s fiber insertion, and reconstruction of the functional cementum–PDL–bone interface. Future studies should combine clinically relevant models, dynamic release evaluation, immune and redox monitoring, and functional periodontal regeneration endpoints. Such a shift from material-centered design to microenvironment-informed validation will be essential for advancing periodontal LDDSs toward predictable clinical translation.
